# Understanding willingness to use oral pre-exposure prophylaxis for HIV prevention among men who have sex with men in China

**DOI:** 10.1371/journal.pone.0199525

**Published:** 2018-06-21

**Authors:** Xia Wang, Adam Bourne, Pulin Liu, Jiangli Sun, Thomas Cai, Gitau Mburu, Matteo Cassolato, Bangyuan Wang, Wang Zhou

**Affiliations:** 1 Wuhan Centers for Disease Prevention & Control, Wuhan, Hubei, China; 2 Australian Research Centre in Sex, Health & Society, La Trobe University, Melbourne, Australia; 3 Sigma Research, London School of Hygiene and Tropical Medicine, London, United Kingdom; 4 AIDS Care China, Kunming, Yunnan, China; 5 AIDS Care China, Nanning, Guangxi, China; 6 International HIV/AIDS Alliance, Brighton, United Kingdom; 7 Division of Health Research, Lancaster University, United Kingdom; Agencia de Salut Publica de Barcelona, SPAIN

## Abstract

**Background:**

Oral pre-exposure prophylaxis (PrEP) is recommended as an additional prevention choice for men who have sex with men (MSM) at substantial risk of HIV. The aim of this study was to evaluate the extent, and reasons, for MSM’s willingness to use oral PrEP in Wuhan and Shanghai, China.

**Methods:**

Between May and December 2015, a cross-sectional survey was conducted among 487 MSM recruited through snowball sampling in physical locations frequented by MSM and through social media applications. Exploratory factor analysis was used to group reasons for being willing or not willing to use PrEP. Chi-square tests were used to explore bivariate associations between groupings of reasons for being willing or unwilling to use PrEP, and key sociodemographic and sexual-behavioral characteristics of MSM.

**Results:**

Overall, 71.3% of respondents were willing to use PrEP. The most commonly reported reasons for being willing to use PrEP were preventing HIV infection (91.6%), taking responsibility for own sexual health (72.6%) and protecting family members from harm (59.4%). The main reasons for being unwilling to use PrEP were being worried about side effects (72.9%), the necessity of taking PrEP for long periods of time (54.3%) and cost (40.4%). Individual characteristics that influenced the type of reasons given for being willing or unwilling to use PrEP included being married to a woman, having a regular sex partner, rates of condom use with regular and casual sex partners, and the number of casual sex partners.

**Conclusion:**

The introduction of PrEP in China could benefit from promotion campaigns that emphasize its role in preventing HIV infection, in taking responsibility for own sexual health, and in protecting family members from potential harm. To reduce uptake barriers, it will be essential to provide accurate information to potential PrEP users about the mild and short-term nature of side effects, and the possibility of taking PrEP only during particular periods of life when the risk of HIV exposure might be highest.

## Introduction

Human Immunodeficiency virus (HIV) is a significant cause of global mortality and morbidity [1]. In China, unprotected sex has been the dominant route of HIV transmission since the beginning of the epidemic. The proportion of new cases of HIV infection attributable to sexual transmission has increased in recent years, from 78.7% in 2011 to 94.5% in 2015 [2, 3]. In this context, men who have sex with men (MSM) are a key population at risk of HIV [4], similar to other countries with concentrated HIV epidemics [5]. The most recent estimates suggest that there are between 5–10 million MSM in China [6]. HIV incidence among this population has been reported to be as high as 6.78 per 100 person-years (PY) [7], which is higher than that reported among MSM in other countries such as France (3.8/100 PY) [8] and Thailand (5.9/100 PY) [9].

The high HIV incidence among MSM in China is partly attributable to a high prevalence of unprotected anal intercourse [10, 11] including among HIV positive MSM who are unaware of their status [12]. Despite the availability of various HIV prevention methods, such as condoms and lubricants, testing and treatment for sexually transmitted infections (STI), and linkage to treatment and care for people diagnosed with HIV in China [3], a significant HIV prevention need remains. HIV incidence density among MSM in China is among one of the highest globally [4]. Despite high levels of awareness about HIV and its prevention among Chinese MSM, condom use during anal intercourse has remained sub-optimal [10, 13]. In this context of irregular condom use, and high HIV incidence, it is critical to examine new HIV prevention options for MSM in China.

Oral HIV pre-exposure prophylaxis (PrEP) is a promising biomedical HIV prevention approach in which HIV negative individuals take an oral antiretroviral medication daily to prevent HIV. Several clinical trials have demonstrated the efficacy of oral PrEP for HIV prevention among groups at substantial risk of HIV, including MSM [14–17]. In these trials, adherence was closely associated with the effectiveness of PrEP in preventing HIV [18]. Subsequently, the World Health Organization recommended offering oral PrEP as an additional prevention choice for people at risk of HIV infection as part of a combination HIV prevention strategy [19]. PrEP has already been approved for use among MSM in the United States, France, South Africa, Brazil, and several other countries [20].

While PrEP has proved to be effective in preventing HIV transmission, it is important to better understand how it can be implemented within combination HIV prevention programs based on local contexts. In China, a seminal study conducted in 2012 showed that MSM in Beijing had low levels of awareness of PrEP (11.2%) but had a high level of willingness to use it (67.8%) if it were made available [21]. Similar findings have been reported in a recent review [22] that found a need to provide widespread and accurate information about PrEP to MSM globally.

Beyond understanding awareness levels, formative research is required to identify sub-populations of MSM willing to use PrEP as part of comprehensive prevention efforts, and in what ways they can be supported to access it. In addition, it is essential to identify ways in which MSM can be empowered to make informed decisions on if, how, and when to use it as part of a package of interventions to protect themselves from acquiring HIV. To do so, knowledge, attitudes, and beliefs towards PrEP should be understood, including motivators and barriers to potential utilization.

In order to respond to this imperative, we conducted this study among MSM in Wuhan and Shanghai, with an aim of determining their willingness to use oral PrEP, and the underlying reasons influencing such willingness. In addition, the study sought to identify demographic factors and sexual behavior characteristics associated with willingness to use PrEP among this population.

## Methods

### Study design

The study comprised a cross-sectional survey conducted between May and December 2015 in the cities of Wuhan and Shanghai, which were selected based on their geographical location (Central and Eastern China respectively), the economic status of their residents, and their large MSM populations. Recruitment was conducted by MSM volunteers who were trained by experienced researchers from the London School of Hygiene & Tropical Medicine, and from the Wuhan Centers for Disease Control (CDC).

### Respondent recruitment and survey administration

Recruitment occurred via two methods: (1) three rounds of face-to-face snowball sampling at physical spaces frequented by MSM, such as parks, bathhouses, and bars; and (2) via messages sent over social media applications (WeChat, QQ). Eligibility criteria included: (1) being a man who had sex with men in the previous 12 months; (2) living in Wuhan or Shanghai; (3) being aged 18 years or older; (4) having never received a positive HIV diagnosis; and (5) being willing to sign a consent form. Respondents were given the choice of completing either a paper-and-pencil survey onsite, or an electronic survey online. Both versions of the survey included the same set of questions and information. Following completion of the survey, a small stipend was provided to respondents in Wuhan (30 RMB) and Shanghai (50 RMB). Stipend amounts differed due to higher general costs of living in Shanghai.

### Procedures for paper-and pencil survey onsite

After the initial contact and screening of potential participants, eligible respondents were provided with a choice to either fill out the survey onsite at the time of initial contact or to do so at a future date. Those who chose to fill out the survey at a future date were scheduled for a face to face appointment based on their availability. Before completing the survey, all respondents were provided with a detailed overview of the study, and asked to provide written informed consent. To reduce the chance of potential confidentiality breaches, respondents were permitted to use nicknames when signing the informed consent forms. Filling out the survey form took an average of 30–40 minutes.

### Procedures for responding to the electronic survey online

Respondents who preferred to respond to the online version of the survey were identified in MSM physical or social spaces or through social media applications and provided with a link to the web address hosting the survey, a unique access code, and a password. The landing page, which provided information regarding the study itself, gave access to a series of screening questions, to assess respondents’ eligibility to participate in the study. Eligible respondents were then required to provide consent electronically, after which they gained access to the full survey.

### Survey design

The survey examined knowledge, attitudes and beliefs pertaining to PrEP, and to identify potential reasons for being willing to use it, or not. It consisted of 6 sections, covering demographic characteristics, HIV status, sexual risks and practices, awareness of and willingness to use PrEP, common concerns regarding potential PrEP use, and preferred ways of accessing PrEP. The survey was initially developed in Chinese then translated in English to enable non-Chinese literate team members to provide inputs and comments, and finally back- translated in Chinese by a different translator to avoid bias. To ensure that the survey items were clear, concise, and acceptable to respondents, the survey was piloted with 12 MSM and revised based on feedback.

Self-perception of the risk of HIV infection was measured asking respondents to which extent they agreed or disagreed with the following statements; “It is likely I will contract HIV within the next 12 months” and “The sex I have is always as safe as I want it to be” Responses were recorded using a 5-point Likert scale (strongly agree to strongly disagree).

Given that previous studies had reported low levels of PrEP awareness among Chinese MSM [21, 23], respondents were first asked if they had ever heard of or taken PrEP before. They were then all provided with the following definition *“PrEP is a daily medication that people who do not have HIV take to prevent getting infected with HIV*. *PrEP is taken before someone is exposed to HIV*. *While PrEP is not yet available in China*, *it is thought that PrEP will probably be of most benefit to people who perceive themselves to be at a higher risk of contracting HIV at certain points in their lives*. *PrEP could have more optimal benefit if it is used together with other methods of preventing HIV*. *However*, *it may also be useful for people who have experienced difficulty in using condoms consistently*. *PrEP works best if you take it every day and while there can be some side-effects at first (such as nausea and headaches)*, *these generally reduce after a few weeks of use*. *People who take PrEP should have regular sexual health check-ups*, *including HIV testing to ensure the medication is working*.”

To determine willingness to use PrEP, respondents were asked to indicate their level of agreement on a Likert scale (strongly disagree, disagree, neither agree nor disagree, agree, strongly agree) with seven statements, adapted from a willingness to use PrEP scale used in a previous study [24]. The respondents who chose “Strongly agree” or “Agree” were considered willing to use PrEP, all other responses were considered as unwilling to use PrEP.

To understand what are the main reasons for being willing to use PrEP, respondents were provided with a multiple choice question that included the following potential answers (multiple response options were allowed): (1) to prevent me from contracting HIV, (2) I have problems using condoms, (3) to take responsibility for my own sexual health, (4) I have difficulties persuading my sexual partners to use condoms, (5) to feel more in control of my sexual health, (6) to protect my family from potential harm, (7) other reasons (with an option to specify other reasons not listed above, or to state no special reasons).

Similarly, respondents who stated they were unwilling to use PrEP were asked to select one or more of the following options: (1) I can’t afford the cost; (2) I worry about possible side effects; (3) I worry about forgetting to take my medication; (4) I worry about what other people would think of me; (5) I only have one sexual partner, and we are faithful to each other; (6) I always use condoms; (7) I would not want to take a medication for a long period of time; (8) other reasons (with an option to specify other reasons not listed above, or to state no special reasons).

Survey questions and response options were generated from existing literature regarding potential factors influencing willing to use PrEP in other settings, as reported in previous studies [21, 25–27] and reviews [28]. The survey questions and options were also modified based on feedback from the survey pilot (n = 12) to ensure that the survey items were clear, concise, and acceptable to respondents.

### Data analysis

A total of 496 men completed the survey, however nine respondents were removed from the final database due to duplicate, suspicious or ineligible entries. These comprised: having identical survey codes (n = 2); being HIV positive (n = 4); and having had no sex with men in the previous 12 months (n = 3). The final sample comprised 487 eligible respondents.

Descriptive analysis was undertaken to summarize the sexual behavior and demographic characteristics of the sample. The seven possible reasons for being willing to use PrEP and the eight possible reasons for being unwilling to use PrEP were grouped using exploratory factor analysis with oblique rotation given that the reasons were correlated [29]. Only items with factor loadings of 0.6 and above were retained. After factor analysis, chi-square tests were conducted to determine the association between reasons for being willing to use PrEP (or not) and demographic and sexual behavior characteristics. All analyses were conducted using SPSS version 18.0 (IBM Corp).

### Ethical considerations

Stigma and discrimination against MSM and HIV are widespread in Wuhan and Shanghai. Participation in this PrEP study could have potentially exposed MSM in their communities and exacerbated perceived stigma related to sexual orientation. For this reason, robust strategies were employed to protect the confidentiality and privacy of all study respondents. Only voluntarily consenting respondents were interviewed.

Respondents were assured of confidentiality, anonymity, and their right to withdraw. All were provided with details of the organizations carrying out the survey and a list of websites providing information about HIV, testing, and broader sexual health topics. The study protocol and tools were reviewed and approved by the Institutional Review Board of Wuhan CDC.

## Results

### Demographic characteristics

Of the 487 respondents included in the analysis, 44.6% (n = 217) completed the paper-and-pencil survey, while 55.4% (n = 270) completed the online version. Respondents’ age ranged from 18 to 61 years, with a mean age of 27.68 (±7.15 years). Almost a quarter of the sample, 22.4% (n = 109) were married to a woman, and 73.1% (n = 356) had a college or higher level of education.

### Sexual behavior characteristics

More than four-fifths of respondents (81.1%, n = 395) identified as homosexual and 16.2% (n = 79) as bisexual. In the 12 months preceding the survey, nearly one-third of all the respondents (78.9%, n = 384) had receptive anal intercourse with a male partner, and approximately half of all respondents (45.6%, n = 222) reported that they had a regular sexual partner in the previous 12 months. Nearly three-quarters of the men with a regular partner (73.9%, n = 164) believed that their partners were also HIV negative, while 22.5% (n = 50) were unsure of their partners’ HIV status, and 3.6% (n = 8) knew their partner to be HIV positive (i.e. were sero-discordant). Just over half of respondents with a regular partner (51.4%, n = 114) stated that they always used condoms with that partner, while 20.3% (n = 45) reported that they never did. In the month preceding the survey date, respondents met their sexual partners in public squares/parks/toilets (33.5%), gay saunas or bathhouses (21.1%), gay bars (44.8%), or through the internet and mobile dating applications. (48.7%).

### Perceived risk of HIV infection and knowledge about PrEP

Nearly two-thirds (64.5%, n = 314) of respondents agreed or strongly agreed with the statement that it was likely they would contract HIV in the next 12 months. Only 36 respondents (n = 7.4%) agreed or strongly agreed that the sex they had was not always as safe as they would like it to be, with an additional 193 (39.6%) registering uncertainty when presented with this statement. Nearly 1 in 5 respondents (19.1%, n = 93) had heard about PrEP: 31 of these (33.3%) from online information and 34 (36.6%) from friends. The majority of respondents who had heard of PrEP prior to the survey understood that PrEP was a kind of antiretroviral medicine used to prevent HIV infection before engaging in high risk behaviours.

### Reasons for being willing or unwilling to use PrEP

Among the 347 (71.3%) respondents who said they would be willing to use PrEP for HIV prevention if it were made available for use in China, the most commonly selected reasons were: “*It can prevent me from contracting HIV*” (91.6%, n = 318); and, “*To take responsibility for my own sexual health*”, which was selected by 72.6% (n = 252) of respondents. See Table 1 for a full account of selected willingness to use PrEP responses.

**Table 1:** Percentage of respondents reporting each of seven specified reasons for willing to use pre-exposure prophylaxis (PrEP).

Reasons for being willing to use PrEP among all respondents (N = 347; multiple responses permitted).	N	%
It can prevent me from contracting Human Immunodeficiency Virus (HIV)	318	91.6%
To take responsibility for my own sexual health	252	72.6%
To protect my family from potential harm	206	59.4%
To feel more in control of my sexual health	172	49.6%
I have problems using condoms	35	10.1%
I have difficulties persuading my sex partners to use condoms	28	8.1%
Other reasons	14	4.0%

Among the 140 respondents who stated that they were not willing to use PrEP, the most commonly selected reason (by 72.9%, n = 102) was that, “I worry *about possible side effects*”, followed by the reason that “*I would not want to take a medication for a long period of time*”, which was selected by 54.3% (n = 76) of respondents (Table 2).

**Table 2:** Percentage of respondents reporting each of eight specified reasons for not willing to use pre-exposure prophylaxis (PrEP).

Reasons for being unwilling to use PrEP among all respondents (N = 140; multiple responses permitted)	N	%
I worry about possible side effects	102	72.9%
I would not want to take the medication for a long period of time	76	54.3%
I always use condoms	54	38.6%
I worry about forgetting to take my medication	43	30.7%
I can’t afford the cost	42	30.0%
I worry about what other people would think of me	27	19.3%
I have only one sex partner and we’re faithful to each other	26	18.6%
Other reasons	16	11.4%

### Groupings of reasons for willingness to use PrEP or not

Following factor analysis of the seven possible reasons why men were willing to use PrEP (see Table 3), the first two factors extracted accounted for 68.7% of the variance in the matrix, and were interpretable. No other factor had a loading of 0.6 or above on any of the ten items. The first factor loaded at almost 0.6 and above on three items that broadly related to what we termed **‘taking responsibility or control of sexual health’**. These were: ‘*to feel more in control of my sexual health’*; ‘*to take responsibility for my own sexual health*’; and ‘*to protect my family from potential harm*.’ The second factor loaded at almost 0.6 and above on two items related to a concept that was termed **‘problems using condoms’.** These included: *‘I have problems using condoms’*; and *‘I have difficulties persuading my sexual partners to use condoms’*.

**Table 3:** Factor loadings for the first two principal factors of seven specified reasons for willing to use pre-exposure prophylaxis (PrEP).

Reasons for willing to use PrEP	Factor	
	1	2
It can prevent me from contracting Human Immunodeficiency Virus (HIV)	.158	.303
I should take responsibility for my own sexual health	**.791**	.104
To protect my family from potential harm	**.826**	.228
I feel more in control of my sexual health	**.780**	.245
I have problems using condoms	.051	**.688**
I have difficulties persuading my sex partners to use condoms	.187	**.754**
Other reasons	.172	.415

In factor analysis of the eight possible reasons why men were unwilling to use PrEP, the first three components extracted accounted for 65.2% of the variance in the matrix, and were interpretable. Factor loadings for the eight items are shown in Table 4. No other factor had a loading of 0.6 or above on any of the ten items. The first factor loaded at almost 0.6 and above on four items related to a concept that was termed **‘concerns about use of medications’.** These included: ‘*I worry about possible side effects*’; ‘*I would worry about forgetting to take my medication’*; ‘*I would not want to take a medication for a long period of time*’; and ‘*other reasons’*. The second factor loaded at almost 0.6 and above on two items related to **‘perception of insufficient need’** and included**, ‘***I always use condoms’*; and ‘*I only have one sexual partner and we are faithful to each other*’. The third factor loaded at almost 0.6 and above on two items related to ‘**practical concerns of everyday use’** which included: ‘*I cannot afford the cost’*; and ‘*I would worry about what other people would think of me*.’

**Table 4:** Factor loadings for the first three principal factors of eight specified reasons for unwilling to use pre-exposure prophylaxis (PrEP).

Reasons for unwilling to use PrEP	Factor		
	1	2	3
I worry about possible side effects	**.777**	.054	-.236
I would not want to take the medication for a long period of time	**.751**	.002	.075
I always use condoms	.169	**.753**	-.018
I worry about forgetting to take my medication	**.632**	-.012	.026
I can’t afford the cost	.294	-.271	**-.668**
I worry about what other people would think of me	.410	-.071	**.617**
I have only one sex partner and we’re faithful to each other	-.108	**.678**	.152
Other reasons	**-.700**	-.368	.478

### Association between reasons for being willing to use PrEP (or not) and key demographic and sexual behavior characteristics

Tables 5 and 6 display the relationship between willingness to use PrEP and key demographic and sexual behavior characteristics. Compared with respondents whose marital status was single, divorced or widowed, men who were married to a woman were more likely to explain their willingness to use PrEP in terms of **taking responsibility or control of sexual health** (88.0% versus 77.6%; χ^2^ = 4.638; p<0.05). The same pattern was observed with respect of the factor termed **‘problems using condoms’** (22.8% among those who were currently married to a woman versus 12.9% who were not; χ^2^ = 5.027; p<0.05). In addition, men who reported never using condoms with their regular sexual partners were more likely to explain their willingness to use PrEP in terms of **problems using condoms** compared to men who said they always used condoms (39.4% versus 6.3%; χ^2^ = 22.93; p<0.01). The same pattern was observed with respect of condom use with casual male partners (45.5% versus 8.8%; χ^2^ = 16.81; p<0.01).

**Table 5:** Bivariate analysis of factors associated with willingness to use pre-exposure prophylaxis (PrEP).

Variable	Taking responsibility or control of sexual health		χ^2^	*P* value	Problems using condoms		χ^2^	*P* value
	No	Yes			No	Yes		
Age (years, %)*								
≤24	30(22.4)	104(77.6)			117(87.3)	17(12.7)		
25–34	28(19.6)	115(80.4)			118(82.5)	25(17.5)		
≥35	3(6.5)	43(93.5)	5.708	0.058	39(84.8)	7(15.2)	1.237	0.539
**Married to a female partner**								
Single, divorced or widowed	57(22.4)	198(77.6)			222(87.1)	33(12.9)		
Currently married	11(12.0)	81(88.0)	4.638	0.031	71(77.2)	21(22.8)	5.027	0.025
Sex position with other men in the last 12 months*								
Insertive	18(24.0)	57(76.0)			64(85.3)	11(14.7)		
Receptive	20(18.5)	88(81.5)			89(82.4)	19(17.6)		
Both insertive and receptive	29(18.2)	130(81.8)	1.189	0.552	135(84.9)	24(15.1)	0.393	0.822
**Have a regular sex partner**								
Yes	32(20.1)	127(79.9)			137(86.2)	22(13.8)		
No	36(19.1)	152(80.9)	0.052	0.819	156(83.0)	32(17.0)	0.665	0.415

*Variables with missing data

**Table 6:** Bivariate analysis of factors associated with willingness to use pre-exposure prophylaxis (PrEP).

Variable	Taking responsibility or control of sexual health		χ^2^	*P* value	Problems using condoms		χ^2^	*P* value
	No	Yes			No	Yes		
**Condom use with regular partner**								
Always	17(21.5)	62(78.5)			74(93.7)	5(6.3)		
Often/sometimes	12(25.5)	35(74.5)			43(91.5)	4(8.5)		
Never	3(9.1)	30(90.9)	3.450	0.178	20(60.6)	13(39.4)	22.93	<0.001
**Number of casual sex partners in the last 12 months**								
0	19(25.3)	56(74.7)			65(86.7)	10(13.3)		
1–3	35(20.3)	137(79.7)			145(84.3)	27(15.7)		
≥4	14(14.0)	86(86.0)	3.616	0.164	83(83.0)	17(17.0)	0.443	0.801
**Frequency of condom use with casual sex partners in the last 12 months**								
Always	30(20.3)	118(79.7)			135(91.2)	13(8.8)		
Often/sometimes	18(15.9)	95(84.1)			87(77.0)	26(23.0)		
Never	1(9.1)	10 (90.9)	1.436	0.488	6(54.5)	5(45.5)	16.81	<0.001

As shown in the following Tables 7 and 8, men who had regular sexual partners were likely to select responses that related to a **perception of insufficient need** compared to those who did not have a regular sexual partner (60.3% versus 36.4%; χ^2^ = 7.979; p<0.01). The same pattern was observed between those who had no casual sexual partners within the last year compared to those who had (65.0% versus 30.3%; χ^2^ = 9.025; p<0.05). Those who said they always used condoms with casual partners were more likely to indicate responses that comprised the **perception of insufficient need** factor compared to those who said they never did so (58.0% versus 0.00%; χ^2^ = 8.902; p<0.05). Respondents without a regular sexual partner were more likely to select responses that comprise the **practical concerns of everyday use** factor, compared to those that did (53.2% versus 33.3%; χ^2^ = 5.569; p<0.05).

**Table 7:** Bivariate analysis of factors associated with unwillingness to use pre-exposure prophylaxis (PrEP).

Variable	Concerns about use of medications		χ^2^	*P* value	Perception of insufficient need		χ^2^	*P* value	Practical concerns of everyday use		χ^2^	*P* value
	No	Yes			No	Yes			No	Yes		
Age (years, %)*												
≤24	7(13.2)	46(86.8)			25(47.2)	28(52.8)			30(56.6)	23(43.4)		
25–34	8(11.3)	63(88.7)			40(56.3)	31(43.7)			40(56.3)	31(43.7)		
≥ 35	2(18.2)	9(81.8)	0.444	0.801	7(63.6)	4(36.4)	1.536	0.464	6(54.5)	5(45.5)	0.016	0.992
**Married to a female partner**												
Single, divorced or widowed	14(11.4)	109(88.6)			67(54.5)	56(45.5)			67(54.5)	56(45.5)		
Currently married	3(17.6)	14(82.4)	0.549	0.459	7(41.2)	10(58.8)	1.059	0.303	11(64.7)	6(35.3)	0.634	0.426
Sex position with other men in the last 12 months*												
Insertive	6(15.0)	34(85.0)			16(40.0)	24(60.0)			23(57.5)	17(42.5)		
Receptive	3(8.1)	34(91.9)			21(56.8)	16(43.2)			26(70.3)	11(29.7)		
Both insertive and receptive	7(11.7)	53(88.3)	0.885	0.642	35(58.3)	25(41.7)	3.594	0.166	29(48.3)	31(51.7)	4.499	0.105
**Have a regular sex partner**												
Yes	9(14.3)	54(85.7)			25(39.7)	38(60.3			42(66.7)	21(33.3)		
No	8(10.4)	69(89.6)	0.493	0.483	49(63.6)	28(36.4)	7.979	0.005	36(46.8)	41(53.2)	5.569	0.018

*Variables with missing data

**Table 8:** Bivariate analysis of factors associated with unwillingness to use pre-exposure prophylaxis (PrEP).

Variable	Concerns about use of medications		χ^2^	*P* value	Perception of insufficient need		χ^2^	*P* value	Practical concerns of everyday use		χ^2^	*P* value
	No	Yes			No	Yes			No	Yes		
**Condom use with regular partner**												
Always	6(17.1)	29(82.9)			12(34.3)	23(65.7)			23(65.7	12(34.3)		
Often/sometimes	1(6.2)	15(93.8)			9(56.2)	7(43.8)			11(68.8	5(31.2)		
Never	2(16.7)	10(83.3)	1.133	0.568	4(33.3)	8(66.7)	2.463	0.292	8(66.7)	4(33.3)	0.046	0.977
**Number of casual sex partners in the last 12 months**												
0	7(17.5)	33(82.5)			14(35.0)	26(65.0)			28(70.0)	12(30.0)		
1–3	8(11.9)	59(88.1)			37(55.2)	30(44.8)			35(52.2)	32(47.8)		
≥4	2(6.1)	31(93.9)	2.223	0.329	23(69.7)	10(30.3)	9.025	0.011	15(45.5)	18(54.5)	5.479	0.065
**Frequency of condom use with casual sex partners in the last 12 months**												
Always	6(9.8)	55(90.2)			30(49.2)	31(50.8)			31(50.8)	30(49.2)		
Often/sometimes	3(8.8)	31(91.2)			25(73.5)	9(26.5)			18(52.9)	16(47.1)		
Never	1(20.0)	4(80.0)	0.610	0.737	5(100.0)	0	8.902	0.012	1(20.0)	4(80.0)	1.934	0.380

## Discussion

This study reports a high level of willingness to use PrEP (71.3%) among MSM in Wuhan and Shanghai, China. This estimate is within the range of several previous studies among MSM in China, that have reported levels of willingness to use PrEP ranging from 19.1% to 91.9% [23, 26, 30–32]. However, the strength of this study is its in-depth exploration of reasons influencing willingness to use PrEP, and the use of multiple recruitment strategies, including social media applications, to reduce potential recruitment bias. Evidence suggests that over 60% MSM in China seek their sexual partners via the internet and mobile applications, and that this rate is even higher among young MSM [33–36]. Studies from China [36] and elsewhere [37], have shown that internet and app-based partner selection is frequently associated with riskier sexual behaviors. Hence recruitment through these channels can be appropriate for reaching potential PrEP users at substantial risk of HIV.

The results from this study indicate that the predominant motivations for using PrEP among MSM are: 1) to prevent HIV infection, 2) to be more responsible for their own health, and, 3) to protect their family from potential harm. These reasons were reported by 91.6%, 72.6% and 59.4% of MSM who are willing to use it, respectively. For MSM who are unwilling to use PrEP, the main reasons given related to concerns regarding potential side effects, the need to take PrEP medication for a long time, and the fact they were using condoms consistently (reported by 72.9%, 54.3% and 38.6% respectively).

Unsurprisingly, the HIV preventive effect of PrEP was the most common reason given for wanting to use it. This finding is consistent with other studies which have reported wanting to stay HIV negative as a significant motivator for being willing to use PrEP [27, 38].

Our analysis suggests that MSM perceive PrEP as a HIV prevention method that gives them the opportunity to take control of their own sexual health and take—or maintain—responsibility for their families’ health. An additional finding in our study was that the subgroup of MSM who were currently married to women were more likely to be willing to use PrEP for reasons related to taking responsibility of own health, control of sexual health, or to protect their families from potential harm[27]. This subgroup of MSM were also more likely to cite difficulties in using condoms as a reason for being willing to take PrEP. Taken together, these findings suggest that the potential for PrEP to protect the well-being of significant others could be a capitalized on in social marketing interventions targeting MSM, although this could be limited by the extent to which they are openly living as MSM.

Problems using condoms and difficulties in persuading sexual partners to use condoms were common reasons for being willing to use PrEP. In addition, the subgroup of men who reported never using condoms with their regular and casual sexual partners were more willing to use PrEP given problems related to condom use, which is consistent with other studies [26]. Acknowledging and identifying sub-populations of MSM who are facing difficulties in using condoms could provide an opportunity to distinguish those who are at substantial risk, and to provide them with PrEP, while emphasizing that as opposed to condoms, PrEP does not prevent other STIs. For such sub-populations, PrEP provision could form an entry point for STI prevention and treatment and other health services.

Our results suggest that concerns about daily use of medications, including potential side effects, the need to take medication for a long time, and potential problems or worries about adherence are the dominant reasons for not being willing to use PrEP. These barriers have been noted in other studies [21, 38], and their predominance suggests that communicating about the generally mild and short-term nature of side effects and about the possibility of taking PrEP only during particular periods of life, when the risk of HIV exposure is highest will be essential to future PrEP uptake [20]. In addition, exploring convenient dosing forms (e.g. injectable PrEP) or schedules (e.g. based on seasons of risk) could be future options, as suggested by others [20, 25, 39].

Another distinct factor influencing willingness to use PrEP related to perception of insufficient need and practical concerns of everyday use. It is reasonable to anticipate that MSM who are in monogamous sexual relationships and/or those that always use condoms would report that they do not perceive a sufficient need for PrEP. This finding suggests that PrEP programs in the study setting may not be overwhelmed with unnecessary requests if PrEP were made available.

Our findings are consistent with other studies in relation to the negative influence of cost [40] and suggest that eliminating, subsidizing or discriminating user costs based on purchasing ability will be needed as noted in, Myanmar, Kenya and Taiwan respectively [27, 40, 41]. As such, social marketing and health promotion campaigns should be broad based in-order to reach public, community and private sector channels.

While not grouped within a specific factor, 19.3% of those not willing to use PrEP indicated that they would ‘worry what other people will think of me’ were they to so, echoing similar concerns of stigmatization noted in Malaysia and India [25, 38]. To successfully overcome stigma directed at PrEP users, it will be important to emphasize that all current and potential PrEP users, (including MSM, sex workers, injecting drug users, clients of sex workers, negative partners in sero-discordant relationships, young people or other sub-populations at substantial risk) are not being irresponsible, but that on the contrary they are taking responsible steps to remain HIV-negative and prevent HIV transmission. It will also require educating communities in-order to address other intersecting issues such as HIV-related stigma and homophobia, among others [42].

### Limitations

Our study has several limitations. First, due to its cross-sectional design, this study assessed associations rather than causal relationships. Second, we utilized a quantitative design with close ended questions to explore reasons affecting willingness to use, and while useful and based on literature, the fielding of pre-specified reasons may could have limited reasons provided by respondents as influencing their willingness to use it. Third, our sample may not be representative of all MSM in China, given that Wuhan and Shanghai are only 2 out of several large Chinese cities where large MSM communities live. Our sample may not representative of all MSM in China given that the majority (73%) of respondents were well educated, having completed college.

Finally, our survey only examines the reasons why (or why not) MSM in the study setting are willing (or not) to use PrEP for HIV prevention if it were made available. Given the hypothetical basis of this study, it is uncertain if, and what proportions of MSM would actually use it when it is made available. Evidence from several studies suggests that reported willingness does not always predict actual use of PrEP [31, 43] and could be contingent on the infrastructure, communication and user-support available to potential users [22]. Despite these limitations, the level and factors affecting willingness to use it reported in this study will inform future planning PrEP programs [22].

### Conclusions

This study documents potential motivations and barriers of willingness to use PrEP among MSM in Wuhan and Shanghai, China, and shows that a majority of MSM are willing to use it if it were made available. Successfully implementing PrEP in China and elsewhere will require close attention to those factors shown to influence an individual’s willingness to use it, including their perception of utility and the extent to which it might positively impact their sexual health behavior. Similarly, clinical and community based intervention aiming to increase uptake and effective use of PrEP need to be mindful of common concerns relating to the regular use of medications and strive to minimize these wherever possible.
